# High-Level Lanthanide-Doped Upconversion Nanoparticles-Based Aptasensor to Increase Carcinoembryonic Antigen Detection Sensitivity

**DOI:** 10.3390/ma18040796

**Published:** 2025-02-11

**Authors:** Lujun Niu, Qiren Sun, Shijia Wei, Dixiang Gong, Enhui Wang, Yan Chen, Lu Xia, Xingyu Liu, Langping Tu, Long Shao, Hongfei Li, Jing Zuo

**Affiliations:** 1 Key Laboratory of Automobile Materials of Ministry of Education, Department of Materials Science and Engineering, Jilin University, Changchun 130022, China; niulj22@mails.jlu.edu.cn (L.N.); sunqr23@mails.jlu.edu.cn (Q.S.); weisj1622@mails.jlu.edu.cn (S.W.); wangeh24@mails.jlu.edu.cn (E.W.); chenyan1621@mails.jlu.edu.cn (Y.C.); 2 Department of Mechanical Engineering, McGill University, Montreal, QC H3A 0C3, Canada; dixiang.gong@mail.mcgill.ca; 3 State Key Laboratory on Integrated Optoelectronics College of Electronic Science and Engineering, Jilin University, Changchun 130012, China; 4 The 49th Research Institute of China Electronics Technology Group Corporation, Harbin 150028, China; xialu19860505@sina.com (L.X.); hit_liuxingyu@163.com (X.L.); 5 School of Materials Science and Engineering, Changchun University of Science and Technology, Changchun 130022, China; tulangping@cust.edu.cn (L.T.); shaolong@cust.edu.cn (L.S.); hongfeili@jlu.edu.cn (H.L.)

**Keywords:** high-level doping, CEA detection, UCNPs-PDA NP sensor, FRET

## Abstract

Boosting the accuracy and speed of cancer detection is highly desirous in tumor detection, and sensors capable of detecting carcinoembryonic antigen (CEA) have great application prospects in this field. A highly sensitive sensor is constructed based on the fluorescence resonance energy transfer (FRET) with heavily rare-earth-doped upconversion nanoparticles (UCNPs) as energy donors and polydopamine nanoparticles (PDA NPs) as energy acceptors. This sensor detects the fluctuations in CEA molecules via luminescence quenching and recovery resulting from a competitive binding assay between CEA and PDA NPs. The high-level-doped design of UCNPs (i.e., NaYF_4_@NaYbF_4_:1%Tm@NaYF_4_) is beneficial, providing upconversion luminescence intensity that is more than 10 times higher than that of the conventional low-level-doped UCNPs (i.e., NaYF_4_@NaYF_4_:20%Yb, 0.2%Tm@NaYF_4_). The sensor exhibits impressive sensitivity. Specifically, in diluted fetal bovine serum, the detection limit reaches 0.013 ng/mL in the range of 0–1.5 ng/mL (S/N = 3), while the detection limit is 1.38 ng/mL in the range of 1.5–250 ng/mL (S/N = 3). This method has great potential for future applications in the rapid and early diagnosis and treatment of cancer.

## 1. Introduction

As a broad-spectrum tumor marker, carcinoembryonic antigen (CEA) can assist in the diagnosis of many types of cancers [1]. Fluctuations in the level of CEA reflect tumor activity and therapeutic efficacy during cancer treatment; therefore, the accurate detection of CEA levels is critical for oncological treatments at present. To date, numerous methods have been exploited for detecting CEA, for instance, the radioimmunoassay (RIA) [2,3], enzyme-linked immunosorbent assay (ELISA) [4,5], chemiluminescent immunoassay (CLIA) [6,7], and electrochemiluminescence immunoassay (ECLIA) [8,9]. Although satisfactory CEA detection results have been achieved to a certain extent, new strategies are still needed to further improve the sensitivity of CEA detection and achieve better results in this field. Among the diverse upgrading strategies, aptamer sensors constructed based on the fluorescence resonance energy transfer (FRET) [10,11] mechanism have high sensitivity [1,12], low toxicity [13], and good operability [14,15], which can further contribute to the improvement in the sensitivity of CEA detection.

In the many aptamer sensors based on the FRET mechanism, upconversion nanoparticles (UCNPs)–polydopamine nanoparticles (PDA NPs) biosensors have tremendous potential. UCNPs have unique luminescent properties that enable the conversion of low-energy near-infrared (NIR) light into high-energy visible light [16]. As a result of NIR excitation, the use of UCNPs as energy donors [17] in the FRET mechanism minimizes the interference from the autofluorescence of biomolecules and scattered light [18]. Notably, with careful design, nanoscale upconversion materials can achieve a higher quantum yield and luminescence efficiency relative to micrometer materials [19]. Nano-sized materials can be designed as a core–multishell structure, which enables the resistance of a negative-concentration quenching effect or allows tunable excitation to be achieved. All these characteristics are difficult to realize in bulk materials. Furthermore, UCNPs have superb biocompatibility [20,21], low toxicity [22], and resistance to photobleaching [23]. Another component of the FRET mechanism is based on the fact that PDA NPs, when used as energy acceptors, have a broad absorption spectrum [24], covering almost the entire ultraviolet–visible (UV–vis) and NIR regions, which makes them efficient fluorescent quenching agents. Moreover, PDA NPs have good biocompatibility [25], stability [26], and low toxicity [27]. Aptasensors based on a combination of UCNPs-PDA NPs effectively combine the advantages of both, featuring unique optical properties, high sensitivity, excellent biocompatibility, low toxicity, and stability.

Currently, a slew of aptasensors based on the combination of UCNPs-PDA NPs have been reported [28,29]. Nevertheless, there is still room for further improvement. One of the most effective focus points is the optimization of the donor structure of UCNPs. Conventional UCNPs are constructed based on a low-doping strategy; specifically, the optimal concentrations of both sensitizing ions and luminescent centers are limited to low levels (typically, Yb, 20–30%; Er, Tm, Ho less than 2% [30]). Continuing to increase the doping concentration is thought to cause the so-called “concentration quenching effect”, which in turn decreases the luminescence efficiency of the material. In the recent past, remarkable progress has been made in the construction of high-level rare-earth-doped upconversion systems, with the involvement of highly doped Er^3+^ core–shell structure material systems [31,32] and high-level Yb^3+^ doping systems [33,34]. These studies have considerably increased the optimal doping concentration of activators or sensitizers through various means (e.g., high excitation irradiance [35], dye sensitization [36], and inert-shell passivation [32,37]) and consequently enhanced the upconversion luminescence intensity by 1–3 orders of magnitude. In spite of this, pertinent research results have not been promptly applied to the detection of tumor markers, as represented by CEA. Currently, the vast majority of FRET-based assay systems are constructed using conventional low-doped UCNPs [29,38]. It is obvious that the introduction of a highly doped system into the relevant assay would reliably enhance the detection sensitivity of the sensor under the same instrumental testing conditions by improving the luminescence properties (high brightness) of the donor material, and, thus, this would be a scientific issue well worth investigating.

**Figure 1:**
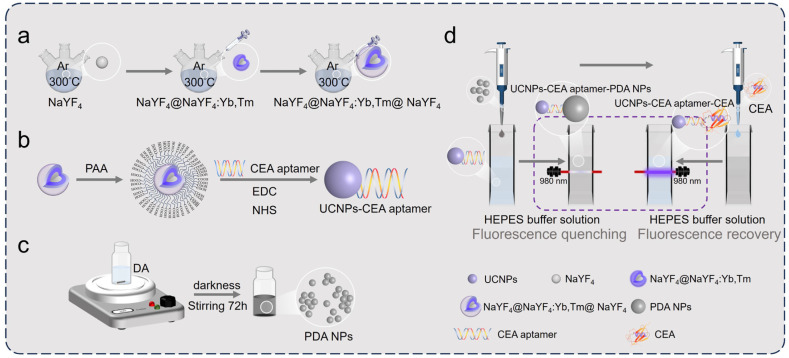
A schematic illustration of the formation of a biosensor for detecting carcinoembryonic antigen (CEA) based on upconversion nanoparticles (UCNPs)-polydopamine nanoparticles (PDA NPs): (**a**) the synthesis process of NaYF_4_@NaYF_4_:Yb,Tm@NaYF_4_ core–shell–shell UCNPs; (**b**) the construction of the UCNP and CEA aptamer combination; (**c**) polydopamine synthesis; (**d**) the fluorescence quenching and fluorescence recovery processes of the detecting system.

Herein, we constructed a PDA sensor based on a highly Yb^3+^-doped core–shell–shell nanostructures as energy donors for the detection of CEA (Figure 1). The synthesized UCNPs were confirmed to have excellent monodispersity and homogeneous dimensions by SEM, TEM, as well as element mapping. FTIR and UV–vis spectroscopy authenticated each other, confirming that the CEA aptamer was modified on the surface of the UCNPs. When PDA NPs were added to the UCNPs-CEA aptamer system, the CEA aptamer bound to PDA NPs to achieve the FRET process, and the fluorescence of the donors was quenched. Subsequently, CEA was added to the system, and specific binding between the CEA aptamer and CEA led to energy donor–acceptor separation, blocking the energy transfer process, and the donor fluorescence was restored. The relationship between the degree of fluorescence signal recovery of the system and the concentration of CEA was used to achieve the quantitative detection of CEA.

## 2. Materials and Methods

### 2.1. Materials and Apparatus

The materials used in this research were RECl_3_·6H_2_O (Re: Y > 99%), oleic acid (OA, 90%), and 1-octadecene (ODE, 90%), purchased from Sigma-Aldrich (St. Louis, MO, USA). Sodium trifluoroacetate CF_3_COONa (90%) was purchased from Macklin (Shanghai, China). RE_2_O_3_ (Re: Y, Yb, Tm > 99%), trifluoroacetic acid (99%), sodium hydroxide (NaOH, 99.9%), 2-morpholinoethanesulphonic acid (MES), 1-(3-dimethylaminopropyl)-3-ethylcarbodiimide (EDC), N-hydroxysulfosuccinimide sodium salt (Sulfo-NHS), 2-[4-(2-hydroxyethyl)piperazin-1-yl]ethanesulfonic acid (HEPES), poly(acrylic acid) (PAA), tris-hydrochloride buffer (PH 8.5), 4-(2-Amlnoethyl)benzene-1,2-diol (Dopamine, DA) (98%), acetone, glycine (Gly), L-ascorbic acid (L-AscH2), L-cysteine (L-Cys), glucose (GLU), bovine serum albumin (BSA), and sodium chloride (NaCl), were purchased from Aladdin (Shanghai, China). Ammonium fluoride (NH_4_F) was purchased from XILONG SCIENTIFIC (Shantou, China). Methanol, ethanol, and cyclohexane were purchased from Sinopharm (Shanghai, China). CEA and CEA aptamer were purchased from Sangon Biotech (Shanghai, China). The fetal bovine serum was purchased from Clark Bioscience (Richmond, VA, USA). All chemicals were used as received without further purification.

TEM, HRTEM images, and element mapping were performed with a Tecnai G2 F20S-TWIN D573 electron microscope (FEI Company, Hillsboro, OR, USA) at 300 kV. The X-ray diffraction (XRD) results were obtained with a Bruker D8-advance X-ray powder diffractometer with Cu Kα radiation (λ: 1.542 Å) (Billerica, MA, USA). The steady-state upconversion photoluminescence (UC PL) spectra were measured by a Zolix DCS300PA Data Acquisition System (Beijing, China). Fourier-transform infrared (FTIR) spectroscopy was performed with a Bruker ALPHA II spectrometer (Billerica, MA, USA). Ultraviolet–visible (UV–vis) absorption spectra were recorded on a Puxi T6 UV–vis spectroscopy spectrophotometer (Beijing, China).

### 2.2. Synthesis Procedures

(1) Preparation of bare core UCNPs

A solvothermal method [39] was used to synthesize the 20 nm bare core of β-NaYF_4_. Typically, 6 mL of oleic acid (OA), 15 mL of 1-octadecene (ODE), and 1 mmol YCl_3_·6H_2_O were added simultaneously to a 100 mL three-necked flask and stirred at 160 °C for 20 min under an argon atmosphere until all the solids dissolved. The solution was then cooled down to room temperature, and 10 mL of methanol solution containing NaOH (2.5 mmol) and NH_4_F (4 mmol) was added and then stirred at 80 °C for 30 min. Subsequently, the solution temperature was raised to 300°C for 90 min under an argon atmosphere. The product was washed with ethanol and centrifuged twice. Finally, the obtained bare core nanoparticles were dispersed in cyclohexane.

(2) Preparation of core–shell-structured UCNPs

Shell precursors were prepared before encapsulating the core with the shell. Y_2_O_3_ (1 mmol) was mixed with 20 mL of aqueous trifluoroacetic acid (50%) and refluxed at 110 °C until it became transparent. The yttrium trifluoroacetate precursor [(CF_3_COO)_3_Y] was obtained. The (CF_3_COO)_3_RE precursor can be obtained using the same method. Next, 1 mmol NaYF_4_:20%Yb, 0.2%Tm active shell was prepared as an example. Then, 1 mmol precursor CF_3_COONa, 0.798 mmol yttrium trifluoroacetate precursor [(CF_3_COO)_3_Y], 0.2 mmol ytterbium trifluoroacetate precursor [(CF_3_COO)_3_Yb], and 0.002 mmol thulium trifluoroacetate precursor [(CF_3_COO)_3_Tm] were added simultaneously to a 100 mL three-necked flask with 6 mL of OA and 15 mL of ODE and dissolved at 120 °C in an argon atmosphere to obtain the NaYF_4_:20%Yb, 0.2%Tm active shell precursor. Then, 0.25 mmol of NaYF_4_ bare cores as seeds, along with 6 mL OA and 15 mL ODE, were added into a 50 mL three-necked flask, and stirred at 300°C for 5 min in an argon atmosphere. We injected 0.6 mmol NaYF_4_:20%Yb, 0.2%Tm shell precursor three times at a uniform rate into this 50 mL three-necked flask with 15 min intervals between the first two times and 45 min for the last reaction. The solution was cooled down to room temperature, washed with ethanol, and centrifuged twice. Finally, the produced core–shell NaYF_4_@NaYF_4_:20%Yb, 0.2%Tm nanoparticles were dispersed in cyclohexane. For the encapsulation of the other shell layers, we followed the above experimental procedure.

(3) Preparation of PDA NPs

A total of 12 mg of DA was weighed, dissolved in 25 mL of Tris-HCl buffer (pH 8.5), stirred at room temperature for 72 h, and protected from light until it turned black. At the end of the reaction, 25 mL of acetone was added to achieve sedimentation, then centrifuged at 12,000 r/min for 15 min. The precipitate was dispersed in 6 mL of pure water at a concentration of 2 mg/mL, then stored at 4°C.

(4) Coupling of UCNPs with CEA aptamer

Firstly, PAA was used to modify the UCNPs. We added 60 mg of UCNPs to 20 mL of water containing 200 mg of PAA and stirred overnight. Then, the mixture was centrifuged at 12,000 r/min for 15 min and washed twice to obtain PAA-modified UCNPs (i.e., PAA-UCNPs). These PAA-UCNPs were dispersed in purified water and stored at 4°C. Next, the coupling of PAA-UCNPs and CEA aptamer was carried out using a cross-linking reaction between the amino and carboxyl groups. We added 5 mg (154 μL) of PAA-UCNPs to 3.5 mL of MES buffer (10 mmol/L, pH 5.5, the same later), followed by adding 500 μL of 5 mg/mL EDC, and finally 500 μL of 10 mg/mL Sulfo-NHS. The resulting mixture was stirred for 45 min and then centrifuged at 12,000 r/min for 15 min. The precipitate obtained was dispersed in 5 mL of MES buffer. Subsequently, 250 μL of 20 nmol/mL CEA aptamer solution was added to the mixed solution obtained above, and the reaction was continued overnight on an agitator. On the following day, the reaction solution was centrifuged at 12,000 r/min for 15 min and washed twice with HEPES buffer (10 mmol/L, pH 7.4), and the precipitate was dispersed in 2 mL of HEPES buffer (10 mmol/L, pH 7.4, 50 mmol/L NaCl) to a concentration of 2.5 mg/mL and stored at 4 °C.

(5) Fluorescence quenching experiment

We added 20 μL of 2.5 mg/mL UCNPs-CEA aptamer (NaYF_4_@NaYbF_4_:1%Tm@NaYF_4_) and different volumes (10, 20, 30, 40, 50, 60, 70, 80, 90, and 100 μL) of 2 mg/mL PDA NP solution to a cuvette, and then the volume was adjusted to 400 μL with 10 mmol/L HEPES buffer (pH 7.4) and incubated at 37 °C for 30 min. The upconverted fluorescence intensity of each sample was measured at the selected PDA NP concentration (0.35 mg/mL) for different reaction durations of 0, 3, 5, 10, 15, 20, 25, 30, 35, 40, 45, and 50 min.

(6) Determination of CEA in buffer solution/diluted fetal bovine serum

To determine CEA in HEPES buffer solution, 50 μL of 2.5 mg/mL UCNPs-CEA aptamer and different concentrations of CEA were added to a cuvette. The mixture was fixed to 825 μL and incubated at 37 °C for 1 h. Then, 175 μL of 2 mg/mL PDA NP was added. The mixture was fixed to 1000 μL and incubated for an additional 40 min. Then, the upconverted fluorescence intensity was measured for each sample solution. In order to investigate the selectivity of the UCNPs-CEA aptamer-PDA NPs sensor to CEA, a series of proteins, sugars, and other interfering substances were selected as replacements for the CEA under the same experimental conditions, and then the upconversion fluorescence was recorded at an 980 nm excitation wavelength. The method for detecting CEA in fetal bovine serum was the same as above, only the HEPES solution was replaced with 50-times-diluted fetal bovine serum. The main chemicals, precursors, and their corresponding concentrations in the above experimental parts are summarized in Table S1 of the Supplementary Materials. All abbreviations are listed in Table S2 of the Supplementary Materials.

## 3. Results and Discussion

**Figure 2:**
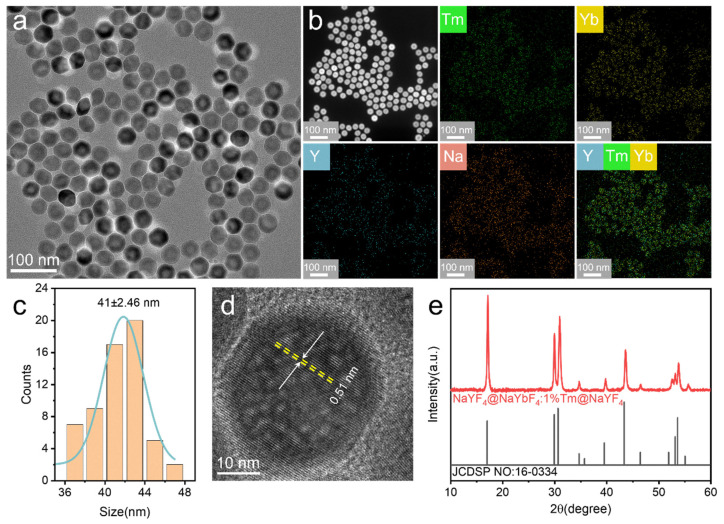
Characterization of synthesized nanoparticles: (**a**) HAADF-STEM image of NaYF_4_@NaYbF_4_:1%Tm@NaYF_4_ core–shell–shell nanoparticles; (**b**) scanning TEM image and element mappings of Tm, Yb, Y, and Na, with an overlap of Y, Tm, and Yb for the sample in panel a; (**c**) the particle size distribution of the sample in panel a; (**d**) high-resolution TEM image of one NaYF_4_@NaYbF_4_:1%Tm@NaYF_4_ core–shell–shell nanoparticle; (**e**) XRD image of NaYF_4_@NaYbF_4_:1%Tm@NaYF_4_ and the standard card (JCDSP No. 16-0334).

Firstly, a series of ytterbium–thulium (NaYF_4_@NaYbF_4_:1%Tm@NaYF_4_ and NaYF_4_@NaYF_4_:20%Yb, 0.2%Tm@NaYF_4_) core–shell–shell nanoparticles were carefully prepared. According to the TEM and SEM images, the bare core (NaYF_4_), core–shell (NaYF_4_@NaYbF_4_:1%Tm and NaYF_4_@NaYF_4_:20%Yb, 0.2%Tm) and core–shell–shell (NaYF_4_@NaYbF_4_:1%Tm@NaYF_4_ and NaYF_4_@NaYF_4_:20%Yb, 0.2%Tm@NaYF_4_) structures exhibited excellent monodispersity and homogeneous dimensions (Figure 2a and Figure S1). As shown in Figure 2a, taking Y@99Yb1Tm@Y (short for NaYF_4_@NaYbF_4_:1%Tm@NaYF_4_) as an example, the structural characteristics of the samples were observed using high-angle annular dark-field scanning transmission electron microscopy (HAADF-STEM). The core–shell–shell sandwich nanostructure was solidly confirmed by the different brightness of the heavy/light lanthanides in the HAADF-STEM imaging (Yb was located in the brighter part of the intermediate shell layer, while Y was located in the darker part of the core and exterior), which matched our design excellently. Additionally, as shown in Figure 2b, the elemental mapping further substantiated that the Yb and Tm were confined to the intermediate shell-layer region. In order to prevent the negative effects of surface quenching, the samples were coated with a 5 nm inert layer of NaYF_4_ as the outermost shell. The statistical results of the particle size revealed that the average diameter of the inner core was ≈ 22 nm, the average thickness of both the intermediate shell layer and the outermost shell layer was ≈ 5 nm, and the average size of the final synthesized Y@99Yb1Tm@Y sample was ≈ 41 nm (Figure 2c and Figure S2). Figure 2d shows the clear lattice fringe, with the d spacing of 0.51 nm corresponding to the (101) crystal plane in the high-resolution transmission electron microscopy (HRTEM) image. The XRD pattern further indicates that the synthesized UCNPs possessed pure hexagonal phases, whose diffraction peaks well matched the standard pattern with card number 16-0334 (Figure 2e and Figure S3).

**Figure 3:**
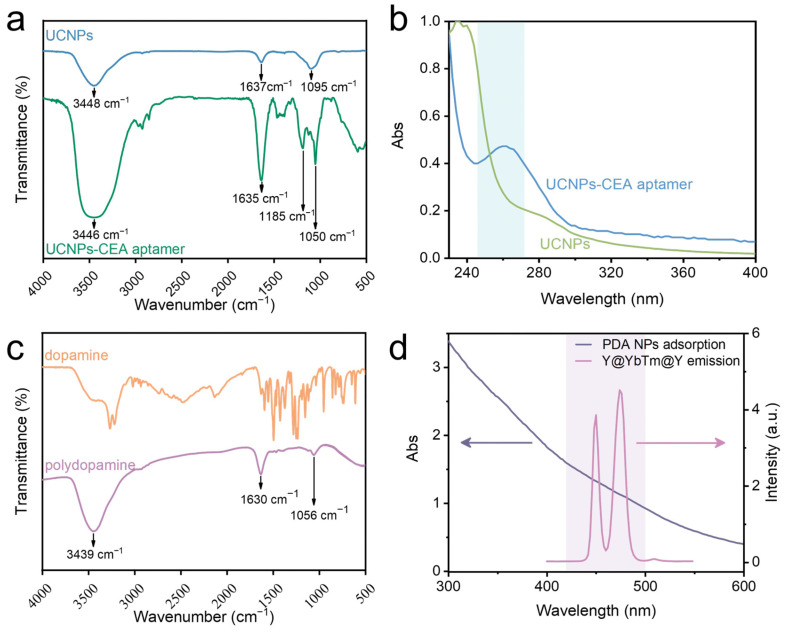
(**a**) FTIR spectra of NaYF_4_@NaYbF_4_:1%Tm@NaYF_4_ (without an organic layer, bule line), and NaYF_4_@NaYbF_4_:1%Tm@NaYF_4_-CEA aptamer (green line); (**b**) UV–vis absorption spectra of NaYF_4_@NaYbF_4_:1%Tm@NaYF_4_ and NaYF_4_@NaYbF_4_:1%Tm@NaYF_4_-CEA aptamer; (**c**) FTIR spectra of dopamine and PDA NPs; (**d**) absorption spectra of PDA NPs, and emission spectra of NaYF_4_@NaYbF_4_:1%Tm@NaYF_4_ UCNPs.

To prove the successful coupling of the UCNPs with the CEA aptamer, we used FTIR and UV–vis spectroscopy for testing. The FTIR spectrum of the UCNPs without organic ligands is shown in Figure 3a (bule line). However, after modification with the CEA aptamer, the FTIR spectrum of the UCNPs-CEA aptamer (green line in Figure 3a) exhibited several characteristic absorption peaks, such as the C–O stretching vibration peaks at 1050 cm^−1^ and 1185 cm^−1^, the stretching vibration peak of C=O at 1635 cm^−1^, and the stretching vibration of the N–H and O–H bonds’ peak at 3446 cm^−1^. The appearance of these characteristic peaks indicated the successful coupling of the UCNPs with the CEA aptamer. To further verify this, we obtained the UV–vis absorption spectra before and after the coupling. Figure 3b shows an apparent characteristic DNA absorption peak (derived from the CEA aptamers) around 260 nm after the coupling of the UCNPs with the CEA aptamer, which further indicated their successful coupling. Subsequently, to confirm that FRET can occur between the coupled UCNPs and PDA NPs, we performed FTIR and UV–vis spectroscopy testing on the PDA NPs (the diameter of the PDA NPs was about 185 nm; the relevant TEM image is displayed in Figure S4). Figure 3c shows that the FTIR absorption peak of the PDA NPs at 1056 cm^−1^ resulted from the stretching vibration of the C–O bonds, the peak at 1630 cm^−1^ as ascribed to the vibration of aromatic structures, and the peak at 3439 cm^−1^ was ascribed to the stretching vibration of symmetric and asymmetric N–H, which suggest that the PDA NPs possessed an aromatic ring structure and properties similar to those of π-conjugated polymers. When PDA NPs are added into the UCNPs-CEA aptamer system, “π–π stacking” interactions occur between the nucleobases of the single-stranded DNA and the aromatic groups of the PDA NPs [40]. This allows the single-stranded DNA (CEA aptamer) to assemble on the PDA surface with strong affinity, and then a combination of UCNPs-CEA aptamer-PDA NPs is formed. Moreover, we tested the absorption spectra of the PDA NPs and the fluorescence emission spectra of the UCNPs; as shown in Figure 3d, they were well-overlapped, which allowed FRET to occur.

**Figure 4:**
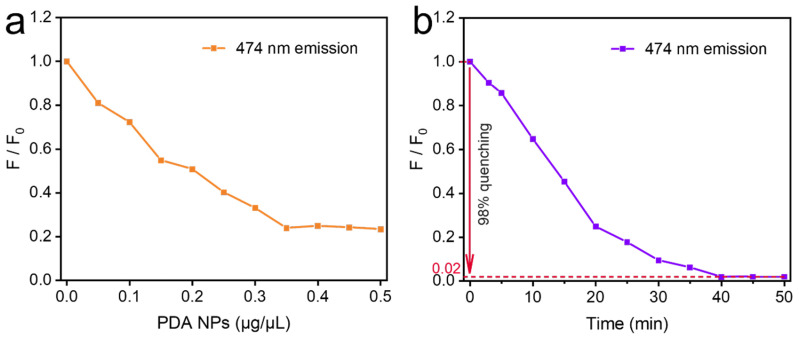
(**a**) Fluorescence quenching of NaYF_4_@NaYbF_4_:1%Tm@NaYF_4_-CEA aptamer system with different concentrations of PDA NPs (the reaction time was fixed at 20 min); (**b**) reaction-time-dependent fluorescence quenching of NaYF_4_@NaYbF_4_:1%Tm@NaYF_4_-CEA aptamer-PDA system (the concentration of PDA NPs was fixed at 0.35 μg/μL).

Next, we optimized two reaction conditions of the FRET system constructed with the PDA NPs and UCNPs (taking Y@99Yb1Tm@Y as an example), i.e., (1) the PDA NP concentration and (2) the donor–acceptor reaction time. Firstly, we fixed the reaction time at 20 min, and different concentrations of PDA NPs were added to the Y@99Yb1Tm@Y-CEA aptamer system. The degree of fluorescence quenching of the system was directly related to the concentration of PDA NPs. Figure 4a shows that the relative fluorescence intensity, F/F_0,_ of the Y@99Yb1Tm@Y-CEA aptamer system gradually decreased with the increase in the concentration of PDA NPs (F represents the fluorescence intensity of the Y@99Yb1Tm@Y-CEA aptamer system at 474 nm with the existence of PDA NPs, and F_0_ is the fluorescence intensity of the system without PDA NPs). When the concentration of added PDA NPs reached 0.35 μg/μL, the fluorescence quenching of the system was saturated. By continuing to increase the concentration of PDA NPs, the value of F/F_0_ remained unchanged, so we determined that 0.35 μg/μL was the optimal concentration of PDA NPs. Based on these results, we further investigated the effect of the reaction time on the fluorescence quenching. As shown in Figure 4b, the value of F/F_0_ gradually decreased with increasing reaction time (the concentration of added PDA NPs was fixed at 0.35 μg/μL) and started to reach a stable plateau at 40 min. Therefore, in the following study, to ensure the completeness of the quenching process, we chose a PDA NP concentration of 0.35 μg/μL and a reaction time of 50 min (guaranteeing a full reaction). In this case, the highest fluorescence quenching of the system reached 98% (Figure 4b), which confirmed the good quality of our designed FRET system.

**Figure 5:**
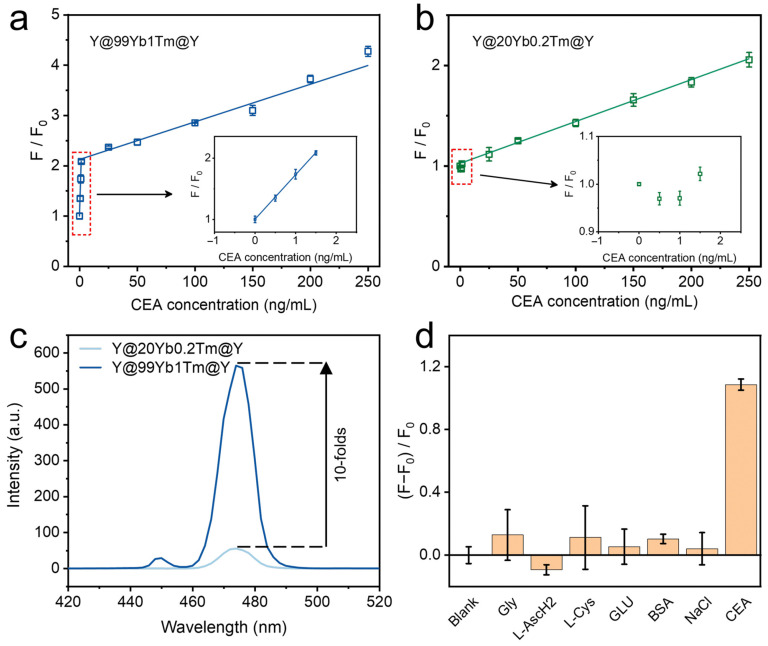
(**a**) Fluorescence recovery of Y@99Yb1Tm@Y-CEA aptamer-PDA NP system with different CEA concentrations; (**b**) fluorescence recovery of Y@20Yb0.2Tm@Y-CEA aptamer-PDA NP system with different CEA concentrations; (**c**) steady-state emission spectra of two types of UCNP under 980 nm laser irradiation (0.80 W cm^−2^); (**d**) relative fluorescence intensity of sensor (0.01 mg/mL) in presence of Gly (1.0 μg/mL), L-AscH2 (1.0 μg/mL), L-Cys (1.0 μg/mL), Glu (1.0 μg/mL), BSA (1.0 μg/mL), NaCl (1.0 μg/mL), and CEA (50 ng/mL). Experiments were performed in HEPES buffer (10 mM, pH 7.4).

Based on the above-mentioned optimization of the two reaction conditions, we further explored the effect of two different representative doping levels of lanthanide ions in the UCNP donor on the sensitivity of CEA detection. The optimal doping concentrations of traditional low-doping structures are 20% Yb^3+^ and <2% Tm^3+^ [41,42]. In recent years, highly Yb^3+^-doped systems have become a research hotspot and have stronger upconversion luminescence brightness compared to the low-doped systems [19,43]. To further clarify the optimal doping concentration relationship between Yb^3+^ and Tm^3+^ in the highly doped system, a series of studies were conducted, which finally confirmed that the optimal luminescence was realized when the doping concentrations of Tm were 0.5%~1% in highly Yb^3+^-doped cases. Therefore, we chose the representative structure of Y@99Yb1Tm@Y to construct the sensor, with the aim of obtaining a larger increase in sensor sensitivity. Additionally, the classical low-doped system Y@20Yb0.2Tm@Y (short for NaYF_4_@NaYF_4_:20%Yb, 0.2%Tm@NaYF_4_) [41,42] was selected as the reference group. Subsequently, we carried out parallel control experiments for both systems. Specifically, we added 0.35 μg/μL PDA NPs to the UCNPs-CEA aptamer-CEA systems. The results showed that the relative fluorescence intensities (i.e., the value of F/F_0_, where F represents the fluorescence intensity of the system at 474 nm in the presence of CEA, and F_0_ is the fluorescence intensity in the absence of CEA) of the systems all gradually recovered as the amount of CEA increased. As shown in Figure 5a, the F/F_0_ value of the highly doped Y@99Yb1Tm@Y system exhibited a segmented linear relationship with the concentration of CEA. When the concentration of CEA was in the range of 0~1.5 ng/mL, a detection limit of 0.0117 ng/mL was obtained (S/N = 3). When the concentration of CEA was in the range of 1.5~250 ng/mL, the detection limit was 1.14 ng/mL (S/N = 3). It is worth noting that this similar segmented relationship has also been reported in previous works [44,45]. On the other hand, the signal variation in the low-doped Y@20Yb0.2Tm@Y system only showed good linearity in the range of 1.5~250 ng/mL, with a relatively large detection limit value (3.23 ng/mL, S/N = 3, as shown in Figure 5b). In that case, it was not difficult to find that the biosensors constructed based on high-doped upconversion systems were significantly more sensitive. The relevant mechanism can be attributed to the high-level-doped donor having superior luminescence performance (e.g., its upconversion intensity was more than 10 times higher than that of the conventional low-doped system, Figure 5c), so that it could better overcome the negative influence of unfavorable factors such as instrumental noise. Additionally, the sensitivity obtained in this work is higher than that of most of the reported methods that also follow the FRET mechanism (as shown in Table 1).

In order to further evaluate the interference immunity of the constructed UCNPs-PDA NP biosensor for CEA detection. We chose several commonly used proteins and small molecules such as Gly, L-AscH2, L-Cys, GLU, BSA, and NaCl as the interfering substances, and all the interfering substance experiments were conducted under the same experimental conditions. The concentrations of all these interfering proteins and small molecules were raised to 1.0 μg/mL; on the contrary, the CEA concentration was limited to 50 ng/mL. As shown in Figure 5d, the fluorescence intensity of the sensor was significantly restored only after adding 50 ng/mL CEA, yet the interfering substances (1.0 μg/mL) did not have a significant effect on the fluorescence of the sensor. This result indicates that our designed sensor has good CEA selectivity.

**Figure 6:**
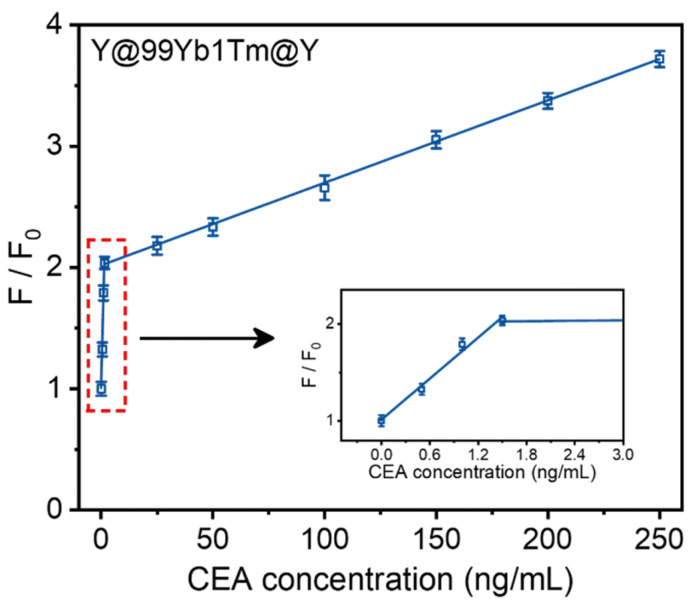
Fluorescence recovery of Y@99Yb1Tm@Y-CEA aptamer-PDA NPs system with different CEA concentrations in diluted fetal bovine serum.

We further evaluated the detection capability of the Y@99Yb1Tm@Y-CEA aptamer-PDA NPs in complex biological matrices, and we conducted tests using 50-fold-diluted fetal bovine serum as the analytical medium. Similar to the results obtained in the HEPES buffer solution, the relationship between the upconverted relative fluorescence intensity (F/F_0_, where F represents the fluorescence intensity of the system at 474 nm in the presence of CEA, and F₀ is the fluorescence intensity in the absence of CEA) and CEA concentrations in diluted fetal bovine serum also showed a linear two-segment correlation. As shown in Figure 6, when the concentration of CEA was in the range of 0~1.5 ng/mL, the detection limit was 0.013 ng/mL (S/N = 3); when the concentration of CEA was in the range of 1.5~250 ng/mL, the detection limit was 1.38 ng/mL (S/N = 3). The similarity between the results above and those in the HEPES buffer suggests that our sensor maintains high sensitivity even in more complex biological matrices. The limits of detection in the diluted serum were slightly higher than those in the HEPES-buffered solutions due to the high complexity of the serum environment. As shown in Figure S5, the fluorescence images of the aptasensor correlated with the CEA concentrations, clearly showing the quenching and recovery processes at different CEA levels. In addition, a standard addition experiment was performed with four diluted serum samples. As shown in Table 2, the recovery rates ranged from 92.2% to 108.7%, and the relative standard deviation (RSD) remained below 6%. These results are acceptable for quantitative determination in biological samples.

## 4. Conclusions

To summarize, we successfully constructed a biosensor (UCNPs-CEA aptamer-PDA NP system) based on the FRET mechanism with high sensitivity in detecting CEA. The utilization of the high-level lanthanide-ion-doping strategy enabled a more than 10-times-higher luminescence intensity than the conventional low-level-doped UCNPs. Benefiting from the enhanced brightness, we achieved a remarkably low detection limit for CEA, which was as low as 0.0117 ng/mL when tested in a HEPES buffer solution and 0.013 ng/mL in diluted fetal bovine serum. Such high sensitivity can meet the common requirement for disease surveillance (≤5.0 ng/mL) and exhibits strong competitiveness compared with numerous counterparts. Given its exceptional sensitivity and reliability, our study provides an alternative sensitive sensor for CEA detection that shows great potential application in the diagnosis of early-stage clinical cancers.

## Figures and Tables

**Table 1:** The reported CEA detection limits of several different fluorescence resonance energy transfer (FRET)-based sensors.

Type of Sensor	Label	Limit of Detection (LOD) (ng/mL)	Linear Concentration Range	Reference
FRET	UCNPs@PDA/AuNPs-CEA aptamer	0.031	0.1 ng/mL to 100 ng/mL	[29]
FRET	Magnetic NPs/UCNPs	0.1	0.1 ng/mL to 40 ng/mL	[14]
FRET	UCNPs/FITC	0.89	0.1 ng/mL to 100 ng/mL	[46]
FRET	UCNPs/carbon nanoparticle	1.0	1 ng/mL to 60 ng/mL	[47]
FRET	Poly(9,9-dioctylfluorenyl-2,7-diyl) dots (PFO dots)/Au-NPs	2.0	0.1 ng/mL to 10 ng/mL	[48]
FRET	UCNPs/Au-NPs	0.02	0.05 ng/mL to 2.0ng/mL	[49]
FRET	Time-gated terbium/quantum dots	3.6	0 ng/mL to 120 ng/mL	[50]
FRET	Quantum dots/gold nanoparticles	0.3	1 ng/mL to 110 ng/mL	[51]
FRET	Quantum dots/tyramide Alexa 594	0.28	0.08 ng/mL to 20 ng/mL	[10]
FRET	UCNPs/PDA NPs	0.0117 or 1.14	0 ng/mL to 1.5 ng/mL or 1.5 ng/mL to 250 ng/mL	This work

**Table 2:** Recoveries of CEA in diluted fetal bovine serum with Y@99Yb1Tm@Y-CEA aptamer-PDA NPs aptasensor (the relative standard deviation (RSD) between three parallel experiments (n = 3)).

Sample No.	Added (ng/mL)	Found (ng/mL)	Recovery (%)	Relative Standard Deviation (%, n=3)
1	0.5	0.482	96.4	2.34
2	1.5	1.467	97.76	4.13
3	10	10.872	108.7	3.5
4	50	46.1	92.2	5.65

## Data Availability

The original contributions presented in this study are included in the article/Supplementary Materials. Further inquiries can be directed to the corresponding author.

## Supplementary Materials

The following supporting information can be downloaded at https://www.mdpi.com/article/10.3390/ma18040796/s1, Table S1: Summary of the consumables, precursors, and concentrations in the Materials and Methods; Table S2: Appendix of all abbreviations; Figure S1: SEM images of (a) bare core NaYF_4_ upconversion nanoparticles (UCNPs); (b) core–shell NaYF_4_@NaYbF_4_:1%Tm UCNPs; (c) core–shell NaYF_4_@NaYF_4_:20%Yb,0.2%Tm UCNPs; (d) core–shell–shell NaYF_4_@NaYF_4_:20%Yb,0.2%Tm@NaYF_4_ UCNPs; Figure S2: (a) The average size of bare core NaYF_4_ particles, measured at 22 ± 1.6 nm. (b) The average size of core–shell NaYF_4_@NaYbF_4_:1%Tm particles, measured at 30 ± 1.48 nm. (c) The average size of core–shell NaYF_4_@NaYF_4_:20%Yb,0.2%Tm particles, measured at 33 ± 1.66 nm. (d) The average size of core–shell–shell NaYF_4_@NaYF_4_:20%Yb,0.2%Tm@NaYF_4_ particles, measured at 46 ± 2.97 nm; Figure S3: XRD patterns of NaYF_4_, NaYF_4_@NaYF_4_:20%Yb,0.2%Tm, NaYF_4_@NaYbF_4_:1%Tm, NaYF_4_@NaYF_4_:20%Yb,0.2%Tm@NaYF_4_ UCNPs, and JCDSP No. 16-0334; Figure S4: TEM image of polydopamine nanoparticles (PDA NPs); Figure S5: Images visualizing Y@99Yb1Tm@Y-PDA NPs aptasensor at different CEA levels.
