# Supplementary material for: High-Level Lanthanide-Doped Upconversion Nanoparticles-Based Aptasensor to Increase Carcinoembryonic Antigen Detection Sensitivity
Source: Materials (Basel). 2025 Feb 11;18(4):796. doi: 10.3390/ma18040796 (PMC11857722; doi:10.3390/ma18040796)
Supplement: Supplementary file 1 [file materials-18-00796-s001.zip › materials-3371055-supplementary.pdf]

## Supplemental Materials for:

# High-Level Lanthanide-Doped Upconversion Nanoparticles-Based Aptasensor to Increase Carcinoembryonic Antigen Detection Sensitivity

Table S1. Summary of the consumables, precursors, and concentrations in Materials and Methods.

| consumables | consumptions | concentrations |
|---|---|---|
| oleic acid (OA) | 6 mL | |
| 1-octadecene (ODE) | 15 mL | |
| $YCl_3 \cdot 6H_2O$ | 1 mmol | |
| NaOH | 2.5 mmol | |
| $NH_4F$ | 4 mmol | |
| methanol | 10 mL | |
| core–$NaYF_4$ | | 0.125 mmol/mL |
| Yttrium trifluoroacetate precursor | 0.798 mmol | |
| Ytterbium trifluoroacetate precursor | 0.2 mmol | |
| Thulium trifluoroacetate precursor | 0.002 mmol | |
| $NaYF_4$:20%Yb,0.2%Tm shell precursor | 0.6 mmol | 0.0476 mmol/mL |
| core–$NaYF_4$ | 0.25 mmol | |
| $NaYF_4$@$NaYbF_4$:1%Tm | | 0.0625@0.15 mmol/ mL |
| $NaYF_4$ shell precursor | | 0.0476 mmol/mL |
| core–shell $NaYF_4$@$NaYbF_4$:1%Tm | 0.125@0.3 mmol (2 mL) | |
| $NaYF_4$@$NaYbF_4$:1%Tm@$NaYF_4$ | | 0.03@0.075@0.1175mmol/mL |
| PDA NPs | | 2mg/ mL |
| UCNPs-CEA aptamer | | 2.5mg/mL |

Table S2. The appendix of all abbreviations.

| Names | abbreviations |
| --- | --- |
| carcinoembryonic antigen | CEA |
| fluorescence resonance energy transfer | FRET |
| upconversion nanoparticles | UCNPs |
| polydopamine nanoparticles | PDA NPs |
| radioimmunoassay | RIA |
| enzyme-linked immunosorbent assay | ELISA |
| chemiluminescent immunoassay | CLIA |
| electrochemiluminescence immunoassay | ECLIA |
| near-infrared | NIR |
| ultraviolet-visible | UV-VIS |
| Oleic acid | OA |
| 1-octadecene | ODE |
| 2-Morpholinoethanesulphonic acid | MES |
| 1-(3-Dimethylaminopropyl)-3-ethylcarbodiimide | EDC |
| N- Hydroxysulfosuccinimide sodium salt | Sulfo-NHS |
| 2-[4-(2-hydroxyethyl)piperazin-1-yl]ethanesulfonic acid | HEPES |
| poly(acrylic acid) | PAA |
| glycine | Gly |
| L-ascorbic acid | L-AscH2 |
| L-cysteine | L-Cys |
| glucose | GLU |
| bovine serum albumin | BSA |
| sodium chloride | NaCl |
| sodium hydroxide | NaOH |
| Ammonium fluoride | $NH_4F$ |
| $NaYF_4$@$NaYbF_4$:1%Tm@$NaYF_4$ | Y@99Yb1Tm@Y |
| $NaYF_4$@$NaYF_4$:20%Yb,0.2%Tm@$NaYF_4$ | Y@20Yb0.2Tm@Y |
| limit of detection | LOD |

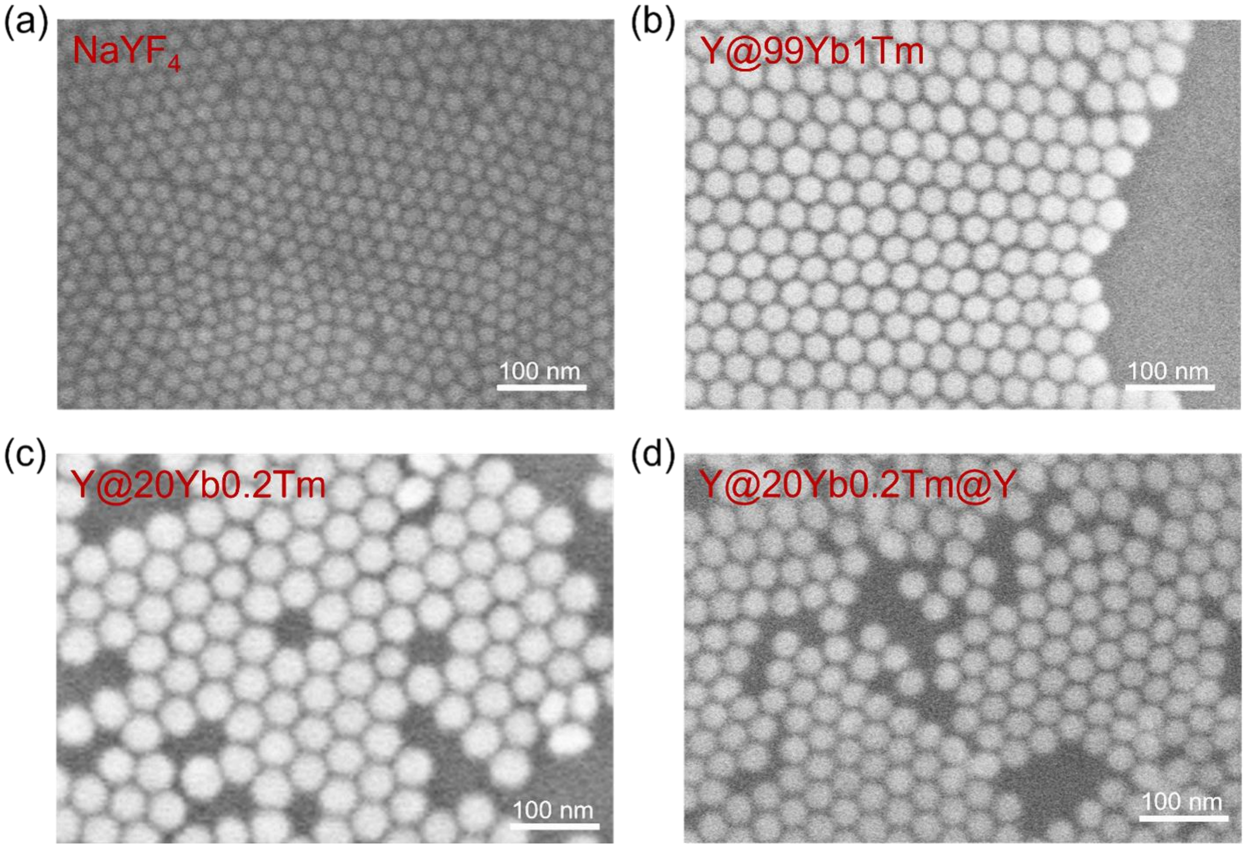


Figure S1. SEM images of (a) bare core $NaYF_4$ upconversion nanoparticles (UCNPs); (b) core–shell $NaYF_4$@$NaYbF_4$:1%Tm UCNPs; (c) core–shell $NaYF_4$@$NaYF_4$:20%Yb,0.2%Tm UCNPs; (d) core–shell–shell $NaYF_4$@$NaYF_4$:20%Yb,0.2%Tm@$NaYF_4$ UCNPs.

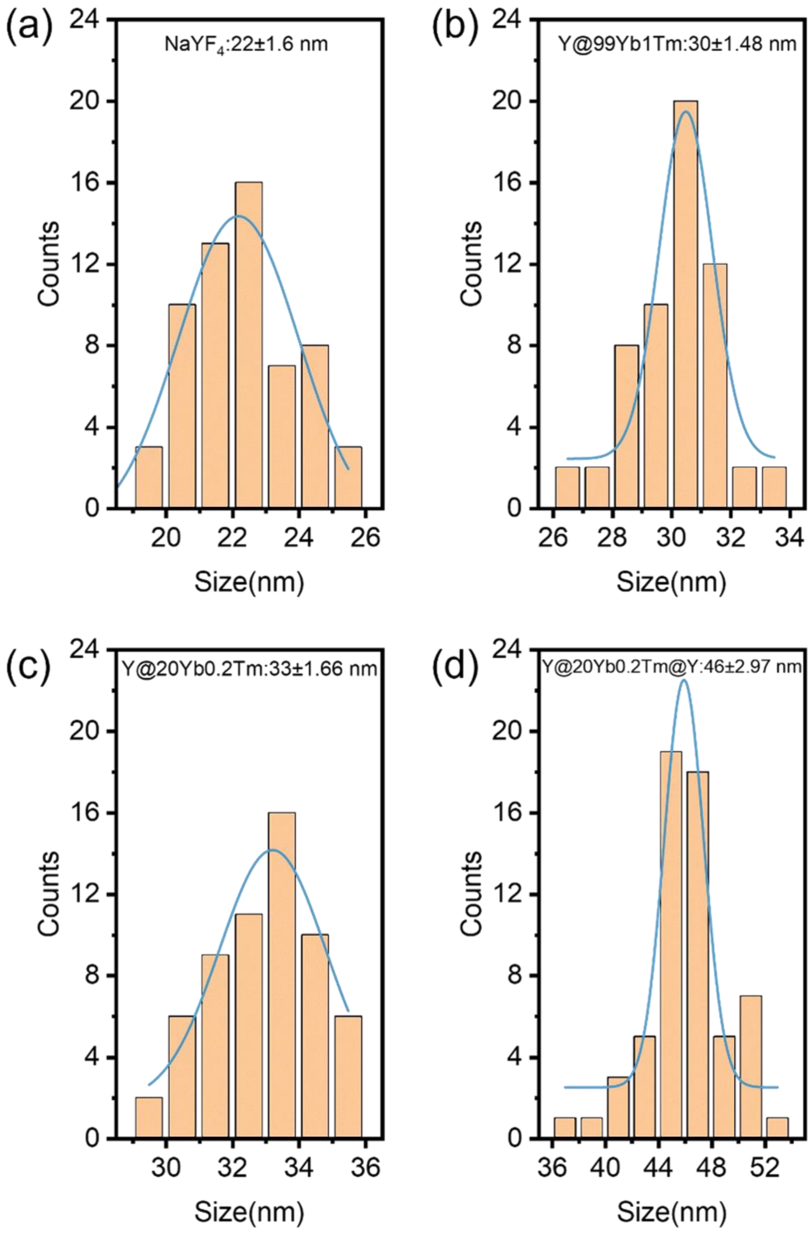


Figure S2. (a) The average size of bare core $NaYF_4$ particles, measured at 22±1.6 nm. (b) The average size of core–shell $NaYF_4$@$NaYbF_4$:1%Tm particles, measured at 30±1.48 nm. (c) The average size of core–shell $NaYF_4$@$NaYF_4$:20%Yb,0.2%Tm particles, measured at 33±1.66 nm. (d) The average size of core–shell–shell $NaYF_4$@$NaYF_4$:20%Yb,0.2%Tm@$NaYF_4$ particles, measured at 46±2.97 nm.

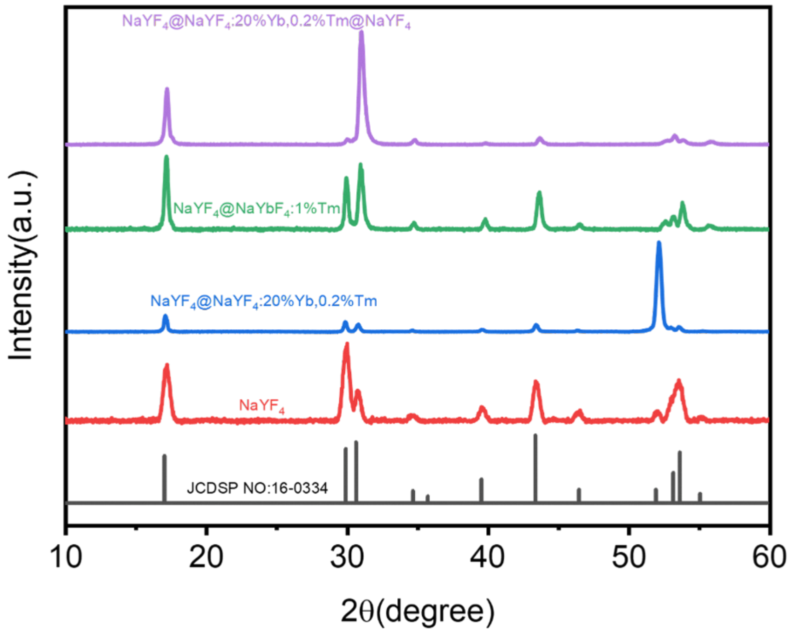


Figure S3. XRD patterns of $NaYF_4$, $NaYF_4$@$NaYF_4$:20%Yb,0.2%Tm, $NaYF_4$@$NaYbF_4$:1%Tm, $NaYF_4$@$NaYF_4$:20%Yb,0.2%Tm@$NaYF_4$ UCNPs, and JCDSP NO:16-0334.

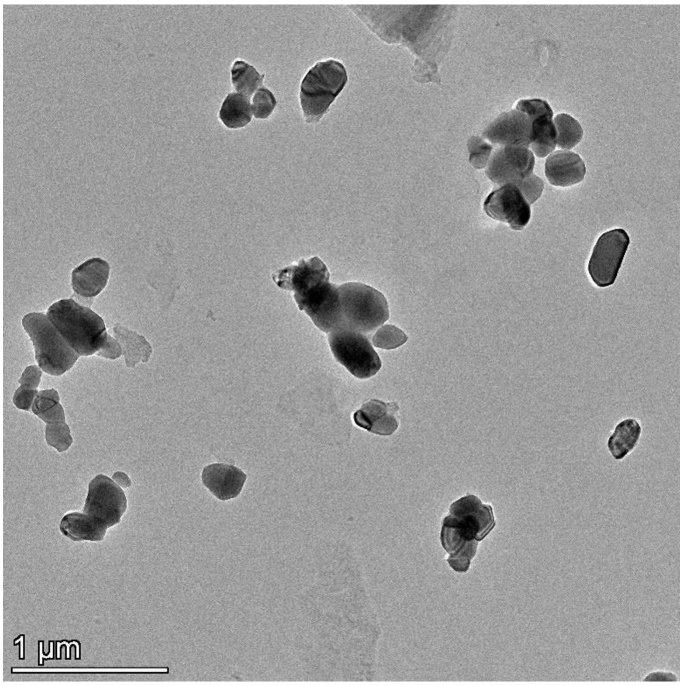


Figure S4. TEM image of polydopamine nanoparticles (PDA NPs).

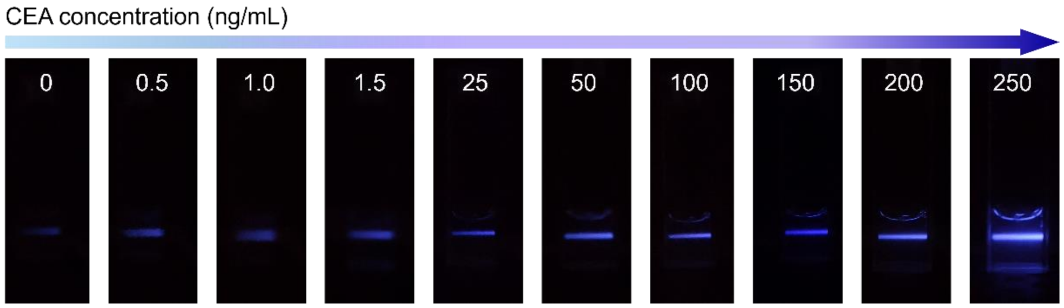

Figure S5. Visible images of the Y@99Yb1Tm@Y-PDA NPs aptasensor at different CEA levels.
